# Intravesical Separation of the Urines

**Published:** 1905-03-18

**Authors:** 


### INTRAVESICAL SEPARATION OF THE URINES.

A little while ago the surgeon who performed a nephrectomy was always harassed by the fear that he might be removing his patient's only kidney, or that the remaining kidney might prove unequal to the task imposed upon it; and so troublesome was this fear that at least one eminent surgeon used to advise opening of the abdomen from the front in order to allow palpation of both kidneys, previous to cutting down through the loin upon the one chiefly or solely affected, notwithstanding the difficulty of forming an opinion as to the functional powers of a kidney, merely by feeling it with the finger through the abdomen. Moreover, it was impossible to tell with certainty in many instances of pyuria or hæmaturia of renal origin which kidney was the diseased one, or whether both were affected, and if so, which was in the worst condition. But now these diagnostic difficulties have been in a very great measure dispelled by the introduction of comparatively simple methods of collecting separately the urine secreted from each kidney. Catheterisation of the ureters, which still has its supporters, is open to the objection that it requires considerable practice before it can be managed with any degree of facility; it is sometimes impossible, and is not always free from danger. For these and other reasons catheterisation of the ureters is an operation of limited applicability. These objections, however, are absent, or nearly so, from some of the methods proposed for intravesical separation of the urines. The instrument designed by Luys, which is probably the best of those at present obtainable, has the advantages of being harmless, easy to use, and trustworthy, whilst its cost is not prohibitive. Theoretic doubts as to its reliability are not sustained in practice. Not the smallest advantage attaching to this instrument is the fact that it requires no previous practice, and it is therefore likely to be of material service to the general practitioner as well as to the consultant.

